# Prognostic value of focal scintigraphic findings in clinically suspected cases of tibial stress fracture

**DOI:** 10.1590/0100-3984.2017.0028

**Published:** 2018

**Authors:** Wagner Castropil, Amisa Guimarães, Carlos Alberto Buchpiguel

**Affiliations:** 1 PhD, MD, Orthopedist at the Instituto Vita, São Paulo, SP, Brazil; 2 MD, Attending Physician for Fleury Group, São Paulo, SP, Brazil; 3 PhD, Full Professor in the Department of Radiology and Oncology, Faculdade de Medicina da Universidade de São Paulo (FMUSP), São Paulo, SP, Brazil

**Keywords:** Tibial fractures, Athletes, Fractures, stress, Radionuclide imaging

## Abstract

**Objective:**

To investigate the correlation between tracer uptake on bone scintigraphy and recovery time in patients with tibial stress fracture.

**Materials and Methods:**

We evaluated two groups of athletes: those with clinical suspicion and a radiological diagnosis of tibial stress fracture (TSF group, n = 21); and those with no symptoms or evidence of fracture (control group, n = 10). All subjects underwent bone scintigraphy and magnetic resonance imaging with a maximum interval of 7 days between the assessments.

**Results:**

Using the region of interest technique, we obtained a quantitative evaluation index, comparing affected and unaffected legs. The mean uptake of ^99m^Tc-MDP was significantly higher in the TSF group than in the control group (2.54 ± 0.77 vs. 1.05 ± 0.11; *p* < 0.001).

**Conclusion:**

In our sample of athletes, determining the bone scintigraphy uptake indices provided an objective method to estimate the appropriate recovery time after a tibial stress fracture.

## INTRODUCTION

In competitive sports, overload injuries are common and may involve the entire locomotor system. When overload injuries affect the bones, they are referred to by various terms, including bone overload, fatigue fracture, and stress fracture. Such fractures are typically seen when abnormal overload occurs within normal bones, promoting bone resorption and subsequently fracture^(1)^.

Stress fractures were first described, prior to the advent of radiography, by the Prussian military doctor Breithaupt in 1855, who introduced the term and described the signs, symptoms, and evolution of stress fractures of the metatarsus^(2)^. In the 1970s and 1980s, when individuals began to increase the frequency and intensity of their exercise regimens, several aspects related to stress fractures were first investigated in the physically active population^(3,4)^.

Plain radiography indicates abnormalities only in more advanced cases or in the later phases of injury, therefore providing limited information and not promoting a deeper understanding of the pathophysiology of stress fractures. However, bone scintigraphy and magnetic resonance imaging (MRI) have provided valuable information regarding bone anatomy and metabolism in patients with stress fractures, alowing subtle and early abnormalities in the structure of bone under stress to be identified^(5,6)^. These two methods may be applied to confirm the clinical diagnosis of a stress fracture, because both have extremely high sensitivity in the detection of fractures or bone abnormalities secondary to a fracture. However, there are limited data in the literature regarding the use of these imaging methods in monitoring the fracture recovery process. A substantial part of the current evaluation method is based on the subjective evaluation of clinical symptoms to estimate whether an athlete is able to return to a regimen involving progressive stress loads.

Because scintigraphy detects early changes in bone metabolism resulting from a stress fracture, it is one of the tests that provide the earliest detection^(7)^. However, one limitation of bone scintigraphy is its lower specificity, due to the fact that a bone injury, even if it does not originate from the fracture, may cause a metabolic bone reaction. Another pitfall of scintigraphy is that old fractures may take time to exhibit metabolic resolution; therefore, positive functional images do not always represent recent fractures^(8)^.

A scintigraphic classification system was devised by Zwas et al.^(8)^, who presented a grading system that correlates the degree of bone involvement with the length of time for which an athlete should be removed from impact activities. That system considers only the extent of the tracer uptake in the affected bone, without taking into consideration its intensity. Determination of the extent of tracer uptake is also based on the quantity of the bone affected in the transverse plane, without incorporating other parameters of extent, such as the degree to which the coronal and sagittal planes are affected.

The introduction of MRI as a complementary diagnostic method for stress fractures made it possible to obtain valuable information regarding the bone anatomy and the adjacent soft tissues in a noninvasive manner. In many cases, MRI can distinguish among distinct pathological conditions that cannot be differentiated with scintigraphy, due to the higher specificity of the former^(5)^. In addition, MRI can demonstrate periosteal edema, mostly in cases of medial tibial stress syndrome (shin splints). However, stress fractures usually present with bone marrow edema and it is sometimes possible to identify the fracture line in severe cases. Fredericson et al.^(9)^ classified tibial stress fractures via MRI and associated the time required for clinical recovery with four degrees of bone involvement. The authors classified tibial stress fractures, according to the MRI findings, as follows:

- Grade 0: normal MRI findings.
- Grade 1: mild to moderate periosteal edema (on T2-weighted images), with normal bone marrow.
- Grade 2: moderate to severe periosteal edema (on T2-weighted images) and bone marrow edema (on T2-weighted images).
- Grade 3: moderate to severe periosteal edema (on T2-weighted images) and bone marrow edema (on T1- and T2-weighted images).
- Grade 4: moderate to severe periosteal edema (T2-weighted images), bone marrow edema (on T1- and T2-weighted images), and a clearly visible fracture line.

However, no correlation has been investigated between this proposed classification and the time to stress fracture recovery. In a recent study, Nattiv et al.^(10)^ determined that a higher MRI grade, lower bone mineral density, and skeletal sites of predominant trabecular bone structures were associated with delayed recovery from bone stress injuries in track and field athletes.

Despite an increasing number of studies in this field, few have correlated bone scintigraphy with MRI. Even fewer studies have investigated a classification to provide guidance regarding the best type of treatment and the necessary recovery time before athletes resume training.

Our study was motivated by the difficulty in obtaining objective criteria for the assessment of the degree of bone involvement in patients with a clinical diagnosis of stress fracture. Such criteria might simultaneously define the ideal resting period and total recovery time needed.

## MATERIALS AND METHODS

Twenty-one patients with clinical findings suggestive of tibial stress fractures (TSF group) were prospectively investigated and underwent bone scintigraphy with technetium-99m-labeled methylene diphosphonate (^99m^Tc-MDP), together with MRI. We also recruited 10 healthy athletes without any clinical signs of medial tibial stress syndrome or stress fractures (control group), who underwent the same protocol. The study was approved by the local research ethics committee, and all participants gave written informed consent.

The TSF group comprised 13 males and 8 females, with a mean age of 31.62 ± 9.39 years. The control group comprised 6 males and 4 females, with a mean age of 29.80 ± 3.94 years. All individuals in both groups were followed for 12 months after the initial examination (including imaging), and all of the individuals in the TSF group underwent the same standard rehabilitation protocol.

### MRI and bone scintigraphy

All MRI examinations were performed in a 1.0 T scanner (Gyroscan T10-NT; Philips Medical Systems, Best, the Netherlands). We obtained T1-weighted sequences and T2-weighted turbo spin echo sequences with fat suppression, in the coronal and axial planes, using a knee coil. The slice thickness was 5 mm, and the field of view ranged from 16 cm (in the axial acquisition) to 20 cm (in the coronal acquisition). The echo time ranged from 12 to 16 ms in the T1-weighted sequences and from 60 to 65 ms in the T2-weighted sequences with fat suppression. The repetition time was in accordance with the number of slices, ranging from 400 to 700 ms in the T1-weighted sequences and from 1800 to 3500 ms in the T2-weighted sequences with fat suppression.

For the diagnosis and follow-up of the patients with stress fractures, we performed three-phase bone scintigraphy using a dual-headed, large-field-of-view, single-photon emission computed tomography gamma camera (Vertex; ADAC, Milpitas, CA, USA). The images were acquired 3-4 h after the intravenous injection of ^99m^Tc-MDP. The dose of radiopharmaceutical was calculated by multiplying patient weight in kg by 11.1-14.8 MBq (0.3-0.4 mCi). The patients were subsequently advised to hydrate aggressively and urinate as necessary. At 3-4 hours after the administration of the radiopharmaceutical, we acquired high-resolution images of the affected and contralateral legs. The metabolic images were acquired under a matrix of 256 × 256 × 16, with an ultra-high resolution, low-energy collimator.

The images were evaluated by two radiologists specializing in musculoskeletal MRI and by two nuclear medicine specialists. The two pairs of specialists, working independently, read the respective images in a double-blind manner, without knowledge of the clinical characteristics of the sample investigated, and reached a consensus. The mean time from the MRI examination to the clinical evaluation was 4 days (range, 2-7 days), and the mean interval between the MRI and the bone scintigraphy was 2 days (maximum, 4 days).

**Figure 1:**
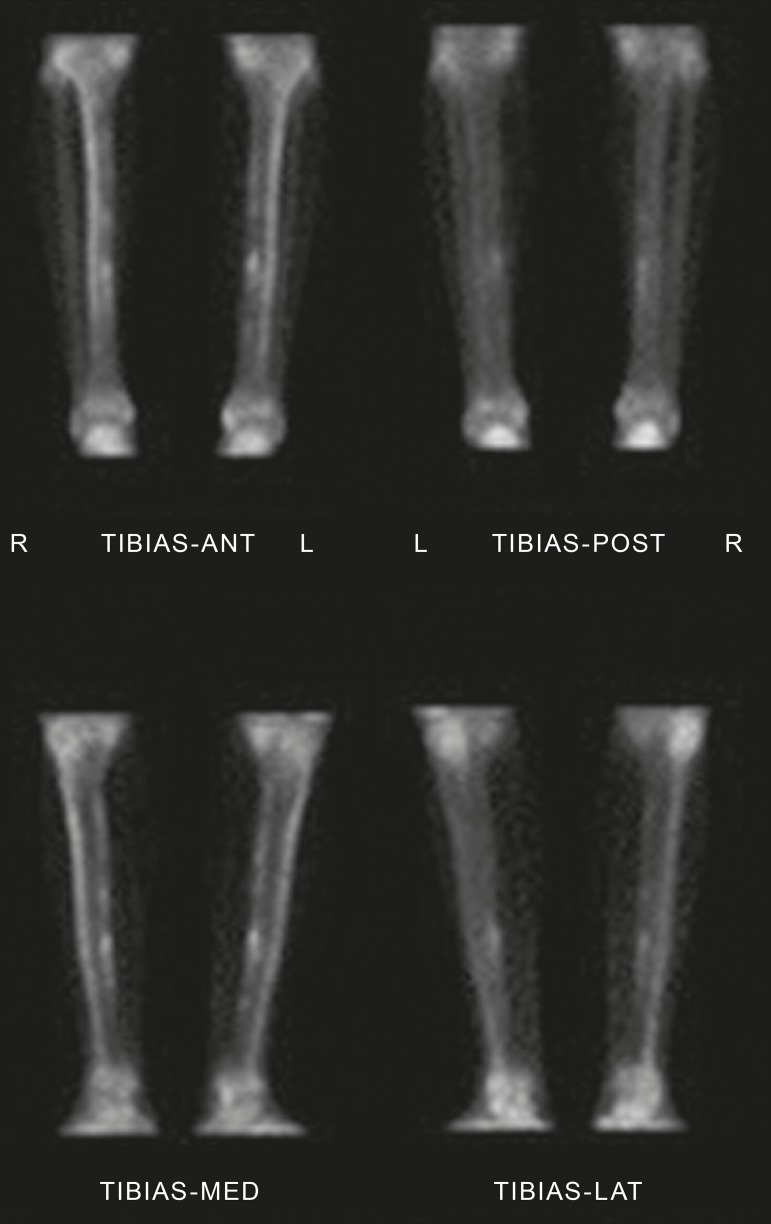
Quantitative analysis of bone scintigraphy.

Quantitative scintigraphy was performed by drawing a region of interest (ROI) around the location of the fracture. The same ROI was used in the contralateral leg as a control area (Figure 1). By recording the total counts within these regions, a quantitative evaluation index (QEI) was obtained by dividing the value for the fractured leg by the value for the control leg according the following formula:

$$\begin{array}{c} \mathrm{QEI}=\mathrm{total}\;\mathrm{counts}\;\mathrm{at}\;\mathrm{the}\;\mathrm{fracture}\;\mathrm{site}/\mathrm{total}\;\mathrm{counts}\\ \mathrm{at}\;\mathrm{the}\;\mathrm{contralateral}\;\mathrm{control}\;\mathrm{site} \end{array}$$

### Treatment protocol

All patients were followed in accordance with a protocol for returning to physical activity, established by Arendt et al.^(11)^, which outlines four progressive phases. In phase I, a walking trial occurred every other day. If the patient was pain free, full ambulation without crutches was initiated. If pain returned at any point in the program, we reinstituted the earlier phase of the program. Once there was pain free walking for 3-5 days, we initiated phase II, which consisted of low-impact weight-bearing activities and muscle rehabilitation. If the patient remained pain free during those activities for 3-5 days, we initiated phase III, which consisted of gradual reentry into a sport-specific activity, which was initially performed on alternate days. The patient performed the sport-specific activity using the onset of pain as a guide for ceasing the activity, pain being rigidly defined as discomfort in the area of the original stress injury to the bone. In some cases, phase III involved very brief episodes of practicing the sport itself. Phase IV included unrestricted practice of the sport, without pain or time modification.

We did not use any other treatment (e.g., pneumatic support, insole treatment, and physiotherapy) that could interfere with the outcome, with the exception of a short course of nonsteroidal anti-inflammatory drugs when clinically indicated. All patients were followed up on a weekly basis during the recovery period and on a monthly basis after they returned to the specific physical activity for at least 12 months after the completion of phase IV. There was no need to immobilize the patients in the initial phase, and crutches were used only in two cases to remove the load for short periods and for pain relief (expected in phase I of the protocol).

The TSF group was comparable to the control group regarding age, weekly training schedule, and principal sports activity.

### Statistical analysis

A comparison between the degree of ^99m^Tc-MDP up-take in bone scintigraphy and the classification according to Fredericson et al.^(9)^ was assessed via the null hypothesis that the groups would have the same mean uptake in the variance analysis, thereby determining the groups of individuals analyzed that would most closely correspond to the Fredericson grades. A multiple linear regression model was also applied, in which the recovery time was the dependent variable and ^99m^Tc-MDP uptake, age, side of the injury, and gender were the independent variables.

## RESULTS

Bone scintigraphy and MRI both demonstrated signs of stress fractures in all of the athletes in the TSF group (sensitivity = 100%). However, both imaging methods also identified nonspecific bone abnormalities in 40% of the asymptomatic (control group) athletes.

**Figure 2:**
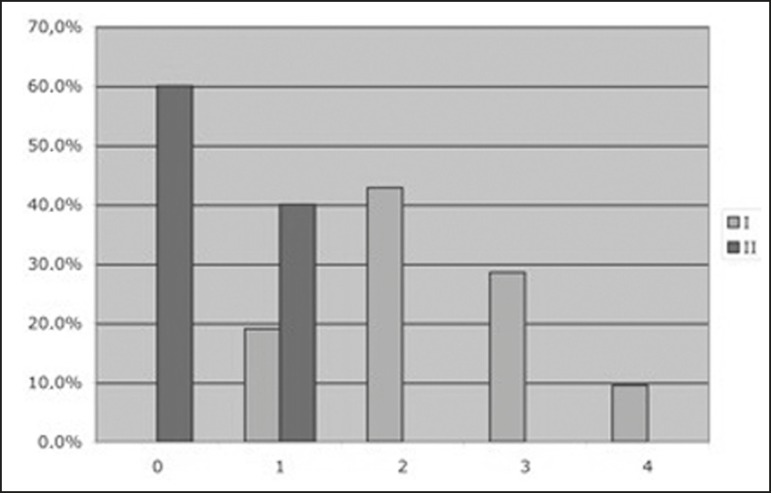
Patient distribution according to the classification system described by Fredericson et al.^(9)^ and the bone scintigraphy ^99m^Tc-MDP uptake QEI.

As shown in Figure 2, the patients in the TSF group were classified, by Fredericson grade, as follows: four patients (19.0%) as grade 1, nine (42.8%) as grade 2, six (28.6%) as grade 3, and only two (9.5%) as grade 4. The patients in the control group were classified as Fredericson grade 0 or 1 (60% and 40%, respectively).

**Figure 3:**
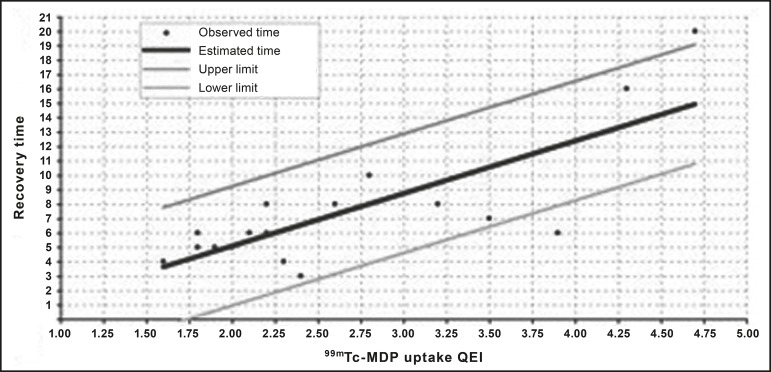
Patient distribution according to the recovery time and the ^99m^Tc-MDP uptake QEI identified from bone scintigraphy.

Figure 3 correlates the recovery time with the ^99m^Tc- MDP uptake QEI for the TSF group, with confidence intervals. We identified a significant correlation between the two variables (R = 0.63; *p* < 0.001). On the basis of those data, we devised a regression equation for estimating the time required for rest or recovery, in accordance with the ^99m^Tc-MDP uptake QEI on bone scintigraphy:

$$\begin{array}{c} \mathrm{Recov}\mathrm{ery}\;\mathrm{time}\;\left(\mathrm{weeks}\right)=-2.24+3.65\times^{99m}\mathrm{Tc}-\mathrm{MDP}\\ \mathrm{uptake}\;\mathrm{QEI}. \end{array}$$

## DISCUSSION

Stress fractures represent an intriguing pathological condition because they affect the bone in a non-traumatic manner. However, they often prevent patients from participating in their sports activities for longer than would be desired. In one interesting study, Stanitski et al.^(12)^ stated that stress fractures are present only in humans, dogs, and racehorses; that is, organisms that are subjected to training with the purpose of maximizing the yield for a specific physical activity.

Plain radiography has been demonstrated to be inefficient in the detection of stress fractures because it often indicates abnormalities only in advanced cases or the later phases of the condition and does not enable determination of the degree of involvement of the bone and surrounding soft tissue^(13)^. With the advent of bone scintigraphy and MRI, it is now possible to obtain valuable information regarding bone anatomy and metabolism in patients with stress fractures^(5,6)^.

Bone scintigraphy provides an early-stage indication of increased degrees of bone remodeling (osteoblastic activity), because of its high sensitivity in the complementary diagnoses of stress fractures^(7)^. In addition, because bone scintigraphy provides an early assessment of bone metabolism changes, it can map out asymptomatic areas that may be transformed into stress fractures^(14)^. It is not unusual for changes in metabolic bone activity to precede structural alterations or changes in radiological density.

Bone scintigraphy has emerged as an efficient method for the diagnosis of pathological bone conditions^(15,16)^, and the literature has confirmed its high sensitivity in the detection of various musculoskeletal disorders^(7)^. Greaney et al.^(17)^ subjectively divided scintigraphic alterations into four levels, in an attempt to quantify the degree of bone involvement. The first scintigraphic classification originated from the study conducted by Chisin et al.^(18)^, who divided the pathological condition into four grades, with no correlation between the classification and the clinical evolution of patients. Zwas et al.^(8)^ presented a classification that correlated four levels of bone involvement with patient recovery time. However, subsequent studies that attempted to apply that classification to predict recovery time failed to demonstrate such an association^(10,19)^.

Increased uptake on bone scintigraphy in asymptomatic individuals is frequently reported in the literature^(10,20)^. That might be explained by the bone remodeling and consequent metabolic increase from the early to late stages of the condition.

With the advent of MRI, studies have demonstrated findings in patients with stress fractures who underwent this novel method. Lee et al.^(5)^ described MRI findings in five patients with a clinical diagnosis and scintigraphic confirmation of stress fractures. The literature indicates that MRI has the same sensitivity as does bone scintigraphy for stress fracture evaluation. However, MRI has the advantage of allowing better evaluation of the soft tissue around the fracture location^(21-24)^. The study conducted by Fredericson et al.^(9)^ is important because it correlates the scintigraphic classification proposed by Zwas et al.^(8)^ with a new classification system using MRI; it also correlates the grades with the prognosis in terms of the amount of time required for the patient to return to sports activities.

Quantitative evaluation, which comprises a method previously investigated in bone tumors^(25)^, has not been previously applied to stress fractures and sports medicine. To our knowledge, there have been no studies retrospectively or prospectively investigating the application of a semi-quantitative evaluation using the ROI technique in relation to stress fracture outcomes. We applied that scintigraphic method with a quantitative analysis in stress fracture patients. This type of evaluation was proposed because we believed that the degree of bone uptake, which represents a direct indicator of the degree of bone remodeling in the affected region, might be related, to a greater or lesser extent, to the severity of the fracture. Although the number of studies in this field is increasing, few studies have aimed to use an index or classification as a guide for determining the best treatment and the minimum time required before athletes resume their sport activities.

We tested the initial hypothesis that greater bone involvement would be related to a longer recovery time and found that the degree to which the bone was affected (anatomically via MRI and metabolically via bone scintigraphy) correlated with the recovery time. Of the rehabilitation protocols investigated in the literature, we chose the one described by Arendt et al.^(11)^, which recommends rehabilitation of the stress fracture in four progressive phases. We selected that protocol because it does not use pre-determined lengths of time for each phase; the periods and phases are modified based on the clinical profile of the patient. Using that protocol, we followed the recovery of each patient for at least six months after their return to the sports activity without restrictions (phase IV). All patients were advised to return to treadmill running because that type of running has been shown to present lower risks^(26)^.

The high sensitivity of MRI and bone scintigraphy might be related to a population bias because we included only athletes who engaged in high-intensity physical activity, had pain upon exertion, and were under clinical suspicion of having a stress fracture. Therefore, it was highly likely that the diagnosis would be a stress fracture. The imaging findings in the asymptomatic (control) group support those in the literature regarding the limited specificity of the imaging methods investigated^(3,19)^. The athletes in the control group were regular practitioners of high-impact physical activity, with consequent periosteal bone alterations identified via bone scintigraphy or MRI. Consequently, they may have been in the initial stage of a stress fracture situation or in the recovery stage of an asymptomatic stress fracture.

We assessed the influence that various factors had on patient recovery time. We found that recovery time did not correlate with age, gender, training intensity, pain location, pain intensity, or the time since symptoms onset. In the assessment of the correlation between the ^99m^Tc-MDP uptake QEI and the recovery time (excluding the influence of other factors), we identified a positive correlation between the proposed index and the recovery time (R = 0.63; *p* < 0.001).

On the basis of the curve that linked ^99m^Tc-MDP uptake with recovery time, a regression equation was formulated to associate the two variables. This approach enabled us to predict the recovery time via the ^99m^Tc-MDP uptake QEI from bone scintigraphy.

It is important to highlight the limitations of our study. The population bias could be considered a limiting factor, as could the small number of patients and the short follow-up time. With a larger number of patients, we would be able to correlate the degree of bone involvement more precisely with the time required to return to activities, as well as with other factors that may result in a worse prognosis related to this pathological condition.

It remains unclear whether the model described in the present study could be applied to other anatomical locations with different biomechanical behaviors. However, it lays the groundwork for future studies involving other common stress fracture locations (e.g., the femur, fibula, and bones of the feet). Further prospective randomized studies are needed in order to validate the use of the proposed method in stress fracture cases, including other bone fracture sites, and to evaluate cases of recurrence with larger samples. Such studies could facilitate the identification of factors that correlate with a worse prognosis, delayed consolidation, or pseudarthrosis.

## CONCLUSION

Bone scintigraphy and MRI were comparable in the evaluation of stress fractures, and both approaches provided quantitative data regarding the injured bone structure. The uptake QEI from bone scintigraphy correlated with the recovery time, which could inform decisions regarding the required amount of time off from physical activities. However, there is a need for randomized controlled studies using the current techniques, with the aim of prospectively and longitudinally assessing the use of the regression equation in a larger patient sample.
